# Maternalism and new imperialism in Russia: “good mothers” for a militarizing state—expectations, implications, and resistances

**DOI:** 10.3389/fsoc.2023.1192822

**Published:** 2023-11-22

**Authors:** Yulia Gradskova

**Affiliations:** Department of Gender Studies, Center for Baltic and East European Studies, Södertörn University, Huddinge, Sweden

**Keywords:** maternalism, Russia's was on Ukraine, traditional values, authoritarian state, militarization

## Abstract

This article explores maternalism in Russia in the context of the contemporary Russian authoritarian state. In particular, I analyze what implications maternalism has for women, mothers, and families on the one hand and how it is connected to the Russian state's new imperial ambitions on the other. I also explore how maternalism is challenged and employed by those resisting state politics, including militarism. Historically, maternalism was used for the analysis of the development of the welfare state in Europe and beyond and for studying women's activism that contributed to significant changes in the state's welfare politics. Maternalism in European history could be seen as “a progressive heterosexual maternal womanhood”; according to Mary Daly, it could be explained as a recognition of the “existence of a uniquely feminine value system based on care and nurturing” and as the assumption that women are performing “a service to the state by raising citizen-workers”. Gender historians of Latin America showed that speaking from the position of a mother was quite important for claiming both the right to be accepted as an equal citizen and the improvement of maternity care, welfare, and living conditions for mothers and children. Furthermore, maternalism was widely used in protests against state militarism, wars, and military dictatorships, not least as a part of the campaign against the Vietnam War or the crimes of the Argentinian military dictatorship. However, maternalism was also widely used by several totalitarian regimes, including fascism and Stalinism. Maternalism was an important political instrument used by the state socialist discourse in order to show the superiority of the “socialist” welfare system over the “capitalist” one and to make this system appear attractive to women from “developing” countries.

## Introduction

Omsk Meat Processing Plant—made in Russia

No way, just like that—made in Russia

Maternity capital—made in Russia

Oxxymiron, September 2022

In the late Soviet Union and during the first period of the history of the Russian Federation, women's organizations that were centered around women's roles as mothers (e.g., Committee of Soldiers' Mothers) became important political actors, demanding the end of Soviet military intervention in Afghanistan and reforms of the army (Caiazza, 2002). However, by as early as the beginning of the 2000s, many such organizations in Russia moved to focusing mainly on the preservation of the memory of their sons and lost much of the potential to be independent pro-democratic actors (Oushakine, 2004). The conservative turn in Russian politics and the widespread propaganda of “traditional values” centered around procreation produced a new version of the state maternalist politics (Bluhm and Brand, 2018, Krafft, 2022, p. 207), while the beginning of Russia's full-scale war against Ukraine on February 24, 2022 made maternalism useful for the activists protesting against the war and mobilization.^1^

Thus, the main aim of this article is to explore how maternalism is interpreted and employed by different actors acting for, but also against, state conservative and imperial politics. In order to do this, I analyze policy documents, Internet presentations, and publications of various organizations, as well as some visual and printed materials connected to the topic of maternalism and parenthood.^2^ Even if “traditional values” are often described as mainly at the heart of activities of religious organizations (Bluhm and Brand, 2018; Stoeckl, 2020), I was interested in a variety of levels and actors; thus, I was looking for state-supported women's organizations, some of which I know through my previous research (Gradskova, 2023). Some of the actors distributing “traditional values” were found by searching the Internet using “traditional values” and “maternity” as keywords.

In order to discuss the connection between maternalism and the new imperialism, I use the theoretical framework developed by Nira Yuval-Davis in her book “Gender and Nation” (1994). She shows that control over marriage, procreation, and sexuality are central to the national agenda, making gender relations central to nationalist projects (Yuval-Davis, 1994, p. 22–23). Using this centrality of gender to nationalist projects described by Yuval-Davis in what follows, I look at the specific case of Russian nationalism that can be called “new imperialism” in connection to nostalgia for the Russian Empire and the Soviet Union and attempts of (re)building an empire with a help of military means.

I start with a short overview of state politics with respect to mothers and children in Russia in connection to the research on ideologies of “traditional values” and “familism”. The second part of the article is dedicated to further exploration of actors participating in the construction of maternalism in the context of “traditional values”. Part three of the article analyzes conservative maternalism's implications for women and mothers in Russia and how maternalism transformed in the context of the Russian war against Ukraine. Finally, the last part deals with resistance to maternalism and the use of maternalism as a resistance strategy.

## The Russian state, “traditional values,” and previous research

While the preoccupation with the falling birth rate and “crises of family” was already at the center of public discussions in Russia in the period of late socialism and perestroika (on expectations of “returning” to “traditions”; see Gradskova, 1997), most serious attempts at state intervention into the sphere of family and maternalism were made during Vladimir Putin's more than 20-year-long term of power, a period characterized by the growth of authoritarianism, nationalism, and militarism. Post-Soviet attempts to improve the birth rate in Russia included a combination of restrictions (a ban on sexual education programs in the mid-1990s; the diminishing number of indications for legal abortion) with attempts to use welfare support for mothers and families—state maternalist politics. The most ambitious among the last was the law on “maternity capital” adopted in 2007, which aimed to reward mothers for giving birth to a second child through contributions to the mother's pension, housing, or education of children. However, as previous research has shown, this initiative was limited in its effect (Isoupova, 2010; Borozdina et al., 2014) and was losing its potential as time went on. In particular, since 2020, maternity capital has started to be paid after the birth of the first child.^3^ In combination with increasingly more aggressive Russian foreign policy (annexation of Crimea, beginning of the war in Eastern Ukraine in 2014, and strengthening of anti-Western rhetoric), the limited impact of maternity capital contributed to the increasing ideologization of all the issues connected to family, intimacy, sexuality, parenthood, and childbearing. For example, in his speech in November 2021, Putin openly declared that demography had become the main problem of the Russian Federation.^4^

It is also important to notice that alongside the law on “maternity capital” from 2007, over the last 20 years, Russia has adopted several laws to contribute to a solution to the problem of the “future of the nation” in its various aspects. While some of these laws were aimed at limiting the accessibility of abortion (including the introduction in 2011 of a 1-week waiting period before an abortion can be performed), others facilitated birth: IVF treatment (since 2006, IVF treatment has been free for both married and unmarried women of reproductive age) and the legalization of surrogate motherhood in 2011. Finally, in 2014, the Russian state started an ambitious program to fight the Soviet legacy of orphanages by financing a foster family program.^5^

However, as previous research has shown, children- and family-centered policies were mainly framed as a part of the defense of “national interests” and not as a respect for the choices of individual women and families (Muravieva, 2016; Gradskova, 2020). Furthermore, these policies simultaneously functioned as politics of exclusion for all “non-desired” parents and families. Indeed, as early as 2012, the Presidential decree banned the international adoption of Russian children by citizens of the United States (the so-called Dima Yakovlev law), while draft laws banning surrogate motherhood services for foreigners and international adoption to citizens of “unfriendly countries” were sent to the Parliament in 2021–2022.^6^ Furthermore, with the infamous “propaganda law” of 2013, LGBTQ+ citizens lost not only their rights to show their identity in public, but also the parental rights of same-sex couples were practically denied (Stella and Nartova, 2015; Kondakov, 2020). Finally, migrant mothers living in Russia did not receive access to the “maternity capital” program, and even those who have acquired Russian citizenship are denied participation in the program if their child was born prior to becoming a citizen of the Russian Federation. It is also important to mention that migrant mothers are usually portrayed in the media not only as “bad mothers” but also as a threat to a good environment for mothering in the country in general.^7^

Researchers studying new conservative ideology have addressed the centrality of family, childbearing, and upbringing of children to Russia's national interests as politics of “traditional values” or “familism” (Edenborg, 2017; Gradskova, 2020). The last term is used less frequently than “traditional values” and more in an academic context; it was introduced in the 1990s by the Moscow State University professor of sociology (internationally known for his anti-gender views) Anatoly Antonov, who proclaimed the importance of returning to the family its central social role^8^ (Antonov, 2019, p. 40; see also Bluhm and Brand, 2018). A family, based on “traditional values” and prized by “familism”, is assumed to be a heterosexual large family founded on patriarchal principles and with an essentialist interpretation of gender roles (Bluhm and Brand, 2018, p. 228; Johnson et al., 2021, p. 509–510). Research has shown, however, that while “traditional values” and “familism” are often used in the media and political documents as “self-obvious”,^9^ their content is subject to contextualization and negotiation by various conservative actors.

Several researchers have shown existing cooperation between “traditional values” ideologists in Russia and anti-LGBTQ and far-right politicians in Europe and America (Edenborg, 2017; Moss, 2017; Sörberg, 2020; Stoeckl, 2020). In particular, pioneering research by Stoeckl has shown close cooperation of actors in Russia with international and transnational conservative networks (2020). Much attention in connection to the distribution of “traditional values” was received by the Russian Orthodox Church (hereafter, ROC), which is often given the central place with regard to the distribution of “traditional values” (Bluhm and Brand, 2018; Krafft, 2022). However, previous research was mainly concentrated on the analysis of legal documents, political speeches, and declarations, while lower levels of the diffusion of “traditional values” ideas through different social networks were explored much less. Furthermore, in spite of the fact that the politics of “traditional values” deal with motherhood and, thus, are part of the politics of women's bodies (see Bordo, 1993), there is not much research on the implications of traditional values for mothers made from gender and feminist perspectives. At the same time, the connection between nationalism and the politics of maternity that was already established by feminist researchers (see Bock and Thane, 1991; Yuval-Davis, 1994) suggests that focus on the maternalism of “traditional values” can make an important contribution not only to understanding the implications of “traditional values” and “familism” for women but also by contributing to the understanding of Russian imperial and military politics.

## “Good mothers” for a strong Russia^10^: maternalism, imperial ambitions, and (state dependent) civil society's actors

While the ideology of “traditional values” is promoted and supported by a rather broad coalition of various actors, the ROC can be seen as having a mediating role and creating presumably “non-political” civic spaces where multiple actors can meet each other and establish or strengthen cooperation. That is why I will start by indicating some such spaces and exploring how maternalism is approached and employed within them. One such space is organized by the Patriarchal Commission on Issues of Family and Defense of Maternity and Children (hereafter, PCIFDMCh).^11^ Up to his death in 2020, the Commission was headed by Archpriest Dmitry Smirnov, known as an active propagandist of “traditional values,” including at the international level (Stoeckl, 2020). The Commission was behind organizing the annual educational conference “Christmas Readings” (*Rozhdestvenskiie chteniia*), and the conference materials can be found on the Commission's website.^12^ These materials indicate that the annual conference dedicated to Christmas began in the mid-1990s and in recent years has become a prestigious place for the meeting of non (or not primarily)-Church-related figures such as university professors, teachers, cultural entrepreneurs, MPs, and representatives of government institutions. For example, the program of the last conference before the pandemic (January 2020) included Natalia Skliarova, pro-rector of the Moscow Pedagogical University, and Margarita Pavlova, TV journalist and member of the Federal Council (Higher Chamber of Parliament; hereafter, FC). Participants of several “Christmas Readings” conferences received official greetings from the state authorities; for example, the 28th conference in January 2020 was greeted by Valentina Matvienko, head of the FC; the participants of the 25th-anniversary conference in January 2017 were sent greetings from Putin himself.

The second meeting space connected to the ROC is *Sanctity of Motherhood* (*Sviatost materinstva*),^13^ the organization created in 2006 by the Christian Orthodox Foundation named after Andrei Pervozvannyi. This organization has a much shorter history than the “Christmas Readings” but is similarly well integrated into the state power structures and civil society. It is headed by Natalia Yakunina, wife of Vladimir Yakunin, a businessman, university professor, supporter of the annexation of Crimea, and high-level politician (Stoeckl, 2020), who also holds annual conferences. The organization's activities enjoy the participation of conservative politicians, including a member of the FC and author of several conservative law drafts, Elena Mizulina. *Sanctity of Motherhood* is a recipient of the grant of the President of the Russian Federation for organizing an annual competition aimed at helping to transform Russian society to become motherhood centered. This competition has several nominations, including for psychologists helping to convince pregnant women to decline abortion and for a medical team to guarantee the best care during pregnancy.^14^ In 2020, Antonov, the “familism” theoretician discussed above, became a member of the council of the organization *Sanctity of Motherhood*.

When discussing the spaces for traditionalist maternalist politics connected to ROC, it is important to mention media platforms and channels. Along with the straightforwardly Christian Orthodox TV channel “Spas” and the website “Pravoslavie.ru”,^15^ it is important to notice “Foma.ru”—a platform presented as a place for those who want to be true believers but have doubts. It was founded by Vladimir Legoyda, a university professor, Press Secretary of the ROC Synod, and, since 2012, a member of the Public Council under the President of the Russian Federation on the rights of the child. *Foma* publishes advice and answers to readers' questions concerning family and the upbringing of children^16^; along with the online version, a “Foma” journal is also published and distributed in Russia and, until February 2022, had a special version for distribution in Ukraine.^17^

Finally, concluding my short overview of spaces for conservative maternalism in connection to the ROC, I want to mention the project “Classical conversations”—a Russian version of the US conservative organizations that advocate for homeschooling. The leader of this organization, Irina Shamolina (the wife of another internationally known personality connected to anti-gender politics, Alexey Komov, along with Antonov, founder of the World Congress of Families—see Sörberg, 2020; Stoeckl, 2020), organizes training for parents, is a frequent guest on orthodox TV and radio programs, and is a former speaker at the “Christmas Readings”.

Now, I will move to another important meeting space for deploying maternalism in the context of “traditional values.” In contrast to the “Christmas Readings” and the “Sanctity of Motherhood”, this space is a part of the Parliament's consultative entity and does not have an explicit connection to the ROC. Indeed, one of the Civic Chamber Commissions is officially titled Commission for Demography, Protection of Family, Children, and Traditional Family Values. The Civic Chamber of the Russian Federation is a high-level consultative entity created in 2005 (Evans et al., 2016) and is tasked with civil society supervision of changes in legislation; it is made up of the representatives of various civic organizations, some of which are nominated directly by Vladimir Putin. The Chamber organizes its work through 20 commissions, including the one I am discussing here. This Commission supervises the implementation of “traditional values” through suggesting and evaluating draft laws and organizing public events in order to communicate with other civil society actors. For example, in 2016, the Civic Chamber's Commission, in cooperation with the Patriarchal Commission on Issues of Family and Defense of Maternity and Children (PCIFDMCh), organized the round table “Traditional Family Values in the System of Higher Education” and published the conference presentations as a book. The book presents opinions by pro-life activists, state officials, and educators; among other things, it insists on several children in each family as a key to Russia's “demographic survival” and decries the death of 1,000,000 “children”^18^ every year due to abortion (2016, p. 78).

Thus, the example of the Civic Chamber shows that while the diffusion of “traditional values” is happening in cooperation with the institutions of ROC, “traditional values” maternalism would be impossible without the work of the various state-controlled civil society organizations, including women's organizations. In order to start discussing in this context the role of the largest of the state-dependent women's organizations—*Women's Union of Russia* (hereafter, WUOR)—I first want to say that from 2019, the position of the Secretary of the Civic Chamber is held by Lidia Mikheeva,^19^ Professor of Law, Vladimir Putin's fiduciary in the 2018 elections, and a member of WUOR.

At first glance, the WUOR appears to be a very different organization to the Christian Orthodox civil society's initiatives: The organization originated in 1941 as a Committee of Soviet Women, and after the end of the Soviet Union, it was transformed into a women's NGO, headed by Ekaterina Lakhova (Buckley, 1997; Gradskova, 2023). While the case of Lakhova's personal transition from a supporter of gender equality to a supporter of anti-LGBTQ legislation is rather well known,^20^ it is less known that in recent years the organization, led by her, went back to close cooperation with the state similar to how it had been during the Soviet period; from 2018, the WUOR has had the official status of a “state-social” (*gosudarstvenno-obshchestvennaia)* organization and uses the colors of the Russian flag on its web pages. The WUOR has member organizations in every administrative unit of the Russian Federation; along with advocating for women's professional education and welfare for children and families, the WUOR member organizations also propagate and organize different events in support of “traditional values”, including educational events for young people and conferences on the “sanctity of maternity, family, and childhood”, with participation from the Orthodox priests and representatives of regional power structures. For example, the WUOR website informs visitors that the WUOR member from the Yaroslavl region organized the “University of motherhood”,^21^ while its member organization from the Riazan region invited women to the seminar “Maternity: the way to happiness”; the latter was attended by the Duma member Mitina. The WUOR website also proudly informs its readers about special medals for mothers^22^ of several children, introduced in some regions by the local governments at the beginning of the 2010s.^23^ Finally, the website also publishes reports on pre-abortion psychological consultations. Indeed, the WUOR member organization from the Khabarovsk region reported in 2018 on a seminar training on the “Psychological consultation of women in the situation of reproductive choice,” supported by the government of the Khabarovsk region.^24^ The article presented pregnant women who had chosen abortion as experiencing psychological problems, a “personality crisis”, and warned women that having an abortion may lead to the worsening of a woman's critical psychological situation.

Separate from the WUOR, *The Union of Women's Forces*^25^ (*Soyuz Zhenskih Sil—*hereafter, UWF) and the *Movement of Mothers of Russia* (*Dvizhenie Materei Rossii*, hereafter, MMR)^26^ are new umbrella organizations that unite several women's groups and organizations of the state-controlled civil society at the local and regional level. The first, the UWF, defines its aim as bringing together active women to solve “economic, investment and ideological problems” and is headed by Inga Legasova.^27^ As well as supporting ideas of female entrepreneurship and discussing issues such as women and design, the organization celebrates “mother heroines” in nominations based on the number of children a woman has brought up and in nominations for “mother of a hero” and “mother of a patriot.”^28^ The second of these new organizations, the MMR, is headed by Valentina Petrenko, a member of the FC, and presents itself as uniting mothers for the “creation of a healthy climate in family and in the working collective” in order to realize the state's politics aimed at support of the family. The organization's activity also aims to “strengthen the institute of family and propagate family values.”^29^ The state plays an important role in the organization's self-description—the colors of the state flag are central to the website's design, while patriotism is often discussed, in particular, with respect to working with youth. The organization partakes in a broad scale of work with mothers in cooperation with the WUOR: Since 2015, it has distributed the pamphlet “Secrets of maternity” to all women who give birth in Russia (Sekrety., 2015). While the practical usefulness of these Soviet-style briefs and the heavily medicalized advice on care for the child and self-care in the first weeks after childbirth can be questioned, it is possible to suppose that this campaign can help these organizations to distribute other information, including those relating to “traditional values”.^30^

Finally, it is important to mention that the state-associated women's organizations are also represented in the international “soft power” structure created by the Russian state in 2015—the *Eurasian Women's Forum*—hereafter, EWF (see Gradskova, 2023). While the first EWF in 2015 allowed the participation of some relatively independent women's NGOs and some gender researchers, the forum of 2018 and, in particular, the last from 2021 were meeting places for those women's organizations in Russia that support state visions of women's social roles and maternalism. The three women's organizations I describe above took an active part in the preparation or realization of the EWF through their leaders or other representatives. For example, Inga Legasova, the leader of the UWF, organized a section in 2018, while Ekaterina Lakhova, the head of the WUOR, made a special YouTube video explaining the role of the upcoming third EWF in 2021.^31^ At variance with the activities of the state-dependent women's organizations for spreading the state vision of maternalism among women in Russia, the EWF is tasked with the important function of positively influencing the international community and female activists abroad (particularly in non-European countries), which it does by emphasizing Russia's advantageous policies toward women (see Gradskova, 2023).

After this overview of the organizations and actors involved, to a greater or lesser extent, in shaping maternalism in the context of “traditional values”, in what follows, I further explore the content of conservative maternalism and its implications for women and connections to the new imperial politics.

## Political implications of the new maternalism for women in Russia—before and after February 24, 2022

From analyzing publications of various actors involved in the deployment of the new conservative maternalism, it is possible to distinguish several important elements of this construction. First, most of the actors concur with the non-acceptance of abortion, viewing it as a violation of public morals and going against the state's interests. Indeed, women's organizations mainly cooperate with the ROC with respect to the training psychologists and convincing women to give birth in place of having an abortion. As a result, pregnant women are undergoing pressure to preserve their pregnancy at any cost and are increasingly becoming the object of attention of professional consultants trained and encouraged by a wide spectrum of organizations, from pro-life to state-dependent women's organizations. At the same time, these organizations also provide women with a Soviet-style discourse, explaining that abortion is also a social problem (Nakachi, 2021) and can be solved through a collaboration of women's organizations, medical professionals, and the state authorities.^32^ For example, according to Lakhova, the head of the WUOR, the National Strategy of Action in Interests of Women in Russia has motherhood as a priority, while women's organizations are expected to help women to realize their social rights as mothers in exchange for giving up the right to abortion.^33^

Second, alongside efforts for preventing abortions, various actors, discussed previously in this article, usually support the idea that the family is a place for happiness, also implying that a happy family is one where there are several children (Bluhm and Brand, 2018, call them large families). It means that a “good mother” should give birth to several children. However, the birth of several children being important to happiness is often followed by its importance to state politics, expressed through state and state-dependent civic organizations' rewards for the most prominent mothers in the form of medals, financial allowances, and honorary nominations. Following Yuval-Davis (1994), it is possible to say that this approach makes nationalism gendered and creates new forms of patriotism constructed through maternalism, focusing on childbirth and the patriotic upbringing of children. On the other hand, women who cannot easily follow these expectations can be assumed to be unpatriotic, ignoring the urgent need for the “demographic survival” of their society (Zadacha Tserkvi, 2016, p. 78) or endangering the nation by allowing the growth of an “alien population” (*ne nashe naselenie*) as the result of migration.^34^

Third, different actors coincide in (heterosexual) marital relationships as grounds for conservative maternalism—only officially married women living in heterosexual families are seen as “good mothers”. Through various means, the defenders of “traditional values” engage in the promotion of lifelong marriage—from educating students about the dangers of unwed motherhood (Zadacha Tserkvi, 2016, p. 77) to organizing “School for brides” (with reference to the WUOR, see the report from the Chelyabinsk region).^35^ In the context of an absence of sex education and widespread domestic violence, the younger generation of women in Russia is expected to prioritize the choice of partner over other interests, including educational and professional ones, and to endure, in some cases, violence from a husband/partner.

However, it is the fourth element of conservative maternalism that has particularly paradoxical consequences: declarations on the importance of parental duties together with expectations that a “good mother” should give all of herself to her children. Indeed, the “traditional values”/familism construction of family often criticizes Soviet maternalism and the state's intrusion into family life, demanding the restoration of the type of family that respects gender hierarchies and has autonomy from the state. According to Antonov, for example, returning to the centrality of the family will help solve other problems of society, including care for the elderly,^36^ while its more radical variant, proposed by the “Classical conversations”, *rasshkolivanie* (liberation from school), supposes that parents will take the role of teachers upon themselves, and children will be “free” from public schools.^37^ However, in spite of the focus on “parents”, who are presented as the best teachers for their own children as they know best all their learning capabilities and problems, the “trainings for parents”, organized by the leader of this organization, Irina Shamolina, seems to assume the mothers' central role in this process. While several actors working for the diffusion of “traditional values” agree on the importance of making fathers' role in the family more significant and trying to involve fathers by using the examples of the Christian Orthodox priests or activists as involved parents,^38^ it is still self-sacrificing motherhood that is at the center of conservative maternalism. For example, an Orthodox psychologist, Vorobieva, addressing questions of stress and tiredness in the context of intensive mothering, hints that for a person who believes in God, it is impossible to say that there is no more energy for the child: “God always gives as much energy as you need.”^39^ While the state-associated women's organizations do not use religious discourse, multiple publications on successful professionals and businesswomen (e.g., the UWF website) suggest that women, if they want, can find time for everything, including being a successful businesswoman alongside being a perfect caregiver to her husband and several children. This can be interpreted as luck of the state's involvement in the programs aimed at better management of work–life balance and welfare for women–mothers. Thus, every mother in Russia should compensate for the risks and insecurity connected to maternity through their own means and solutions.

After this overview of the implications of conservative maternalism for mothers before 2022, it is possible to say that the deployment of maternalism could be seen as one of the forms of preparation for the war. Indeed, constant reminders about demographic crises endangering state security through the possible intrusion of ideological and biological/racial aliens, the expectations of being an involved and self-sufficient mother who does not expect too much, neither from the children's father nor from the state, and the demands of giving birth to several children and bringing them up as patriots obviously contributed to the psychological adjustment to the demands of militarization. In spite of serious violations of women's rights (abortion, equality in the family and in the public sphere), the positive message of the new maternalism—the centrality of the family to society and public (in some cases also material) recognition of maternal work—made many women support “traditional values” and their patriotic and nationalist components.

With the beginning of the Russian full-scale invasion of Ukraine, the “traditional values” started to be diffused more aggressively. Indeed, in May 2022, Vladimir Putin suggested re-establishing the “Mother-hero” nomination and promised to pay a significant sum of money (~1 million rubles) for mothers with 10 or more children. In June 2022, Putin signed a decree making the Day of Love and Fidelity a state holiday,^40^ and the *Mothers of Russia* organization, a part of the UWF, supported it. In July 2022, the Russian Federation's lower house discussed a draft law proposed by Nina Ostanina, head of the Parliamentary Commission for Protection of Family, Women, and Children, aimed at amplifying the 2013 law “on propaganda” to the whole population, stressing the anti-national nature not only of queer but also so-called “childfree” families.^41^ The law was finally accepted on 27 October and entered into force in December 2022. Finally, in July 2023, the law banning medical help to transgender people and depriving them of the right to adopt children or to become foster parents was signed by Putin.^42^

Thus, it is probably not too surprising that most of the organizations deploying conservative maternalism openly supported the Russian state's interpretation of the events in Ukraine and showed their readiness to act in support. *Foma.ru*, for example, published material about providing humanitarian help to people in Donbas,^43^ specifically stressing the role of the ROC.^44^ Some women's “maternalist” organizations supporting the government ignored Russian aggression and the suffering of the Ukrainian people altogether. In particular, the EWF demonstrated to its international audience that it was doing “business as usual” and discussed the successes of female entrepreneurs and women leaders, as well as projects for improving women's health.^45^ However, the WUOR and its regional members openly supported the Russian aggression.

Indeed, the WUOR's webpage published the appeal “Let's help Donbas children,” signed by the head of its organization, Ekaterina Lakhova, several days before 24 February; in August 2022, the WUOR happily noted that help for the children of the Donbas became one of the most supported actions in Russia.^46^ In May 2022, the WUOR published an article authored by the leader of its member organization from Sakhalin, celebrating the fact that Donbas had again become part of the “Russian world”.^47^ Several other reports about support for the Kremlin's politics were also published on the WUOR webpage. For example, a report from the Altai women's organization informed on celebrating the “unification of Crimea with Russia”, where participants also expressed support for the “special operation” in Ukraine and support for the women of Donbas^48^; the article contained information about a competition for patriotic songs in support of the Russian military forces' “struggle against Nazism”.

However, probably the most explicitly militarist document demonstrating support for Russian imperial politics from the women's state-supported organization was the “Appeal of the WUOR”, published on its website on 14 March.^49^ Visually presenting the title of this organization—*C0YUZ* (with a visible “Z”)—the appeal described “the Kyiv regime” as a danger to humanity, accusing it simultaneously of “genocide against Russians,” “war against the Russian world,” the death of many “innocent children,” “terror,” “Nazism,” and “fascism”.

After exploring state maternalism, in what follows, I will give some examples of resistance to the state's conservative maternalism and of the use of maternalism as a strategy of resistance against the new imperial war.

## Resistance to maternalism vs. maternalism as a resistance strategy

The reinforcement of the state propaganda of “traditional values”, presenting an unproblematic synergy between state security interest in increasing the birth rate and the “natural” happiness of maternity, led to several different ways of opposing these politics. As is known, at the beginning of Putin's term and in the context of the possibility of public expression of discontent with the state and regional politics, parents in different parts of Russia took part in demonstrations and the collection of signatures in support of opening more childcare facilities and against long waiting lists to get a place at a kindergarten (Gradskova, 2015). At the same time, several women's organizations in big cities, first in St. Petersburg, organized the defense of mothers who lost their jobs due to violating the law on the preservation of one's workplace during maternity leave (Gradskova, 2017). However, later on, the reinforcement of the propaganda of “traditional values” that increasingly insisted on the centrality of maternity for the life of all women, alongside the growing difficulties for independent women's organizations (Gradskova, 2017), contributed to the transfer of a large part of resistance activity into the virtual sphere. Indeed, several Internet communities approached maternalism from a radical critical perspective, questioning not only the declared state care for mothers and the happiness associated with everyday care work done by mothers but also bringing up the question of the meaning (lessness) of motherhood.

In particular, I want to name here two printed publications (based on blogs started by feminist activists) dedicated to criticism of the “maternity myths” and advising mothers on how to deal with different challenging physical and ideological constraints of motherhood. One of them is the book by Yulia Demakova, Polina Drobina, and Adriana Imzh, #*Happiness of Motherhood* (Demakova et al., 2019). The book is based on the discussions that authors conducted through the Internet portal “V kontakte” over several years. One of the questions posed by the authors to their readers and themselves, for example, sounds like this: “Why is maternity, that can bring so much happiness to a mother, often transformed into a horror film?” In addition to discussing psychological, social, and economic aspects of maternity, the book also includes a great deal of legal information for mothers about their rights in the workplace and rights in respect to the maternity capital governmental program. The second publication that uses a well-known everyday expression for defining a mother's unending duties and responsibilities—titled *You are Mother*—was written by Papudoglo (2016). The book also started as a web blog, led by the author, and contains criticism of the maternalist images of self-sacrificing motherhood.

The beginning of Russia's full-scale invasion of Ukraine led to multiple changes in the country's political and social order, including the emigration of women's rights activists and the closure of independent media that could bring alternative information about the Russian aggression. In this situation, some brave feminist activists, using previous networks, managed to create a new virtual organization: Feminist Anti-war Resistance (FAR), a network of solidarity with Ukrainian victims of the war that protests against Russian aggression. A day will come when the activists themselves will talk more about their organization, but for now, I am mainly using information openly available through the FAR telegram channel (including the newspaper *Zhenskaia Pravda*, distributed there but also available in a PDF format for printing) and articles and several interviews they gave to the media. According to its organizers (most of whom are anonymous), the network is active in ~40 countries, but its activists inside Russia are in constant danger of arrest and police persecution (see Biktimirova and Kravstova, 2022, and an interview with Rossman, 2022). The network's telegram channel coordinates actions of protest and mourning in parallel across different Russian cities and abroad and publishes reports. The participants openly criticize the rhetoric of “traditional values”, stressing that the war and patriarchal authoritarian regime in Russia worsen the situation of women–mothers. For example, in Issue 6 from August 2022, a *Zhenskaia Pravda* author, under the pseudonym Manizha Bulochkina, reported that in 2022, in Russia, fewer children were born than before and that the birth rate was close to that of the 1940s, during the period of the Second World War. This was in spite of the widespread anti-abortion programs and medals for mothers and is connected, among other factors, to the decreasing incomes of families and growing uncertainty with respect to the future (*Zhenskaia Pravda*, 6). Continuing this theme, Issue 8 of the newspaper published another text by the same author that stated that, in spite of conservators' speeches in the Duma on the importance of children, “according to the official data of the Ministry of Finances, one in two families with children (among those who applied for state support—YG) is denied financial support” (*Zhenskaia Pravda*, 8).

However, it is important to note that the FAR not only criticizes state maternalist ideology but also often uses maternalism as a positive instrument for protest against the imperial war. For example, an article signed by Tatiana Zvezdunova in Issue 8 of *Zhenskaia Pravda* used maternalism to protest against the war: Women benefit society in that they bring children to the world and are more responsible for them than men. Because of this bigger responsibility, according to the author, “every mother can stand up and say “This is my child. I brought it to the world. You do not have any right to it'.”

Vladimir Putin's declaration of mobilization on 23 September led to maternalism becoming a more visible way of protesting. Indeed, some organizations of Soldiers' Mothers continued to use maternalism in their work to protect the rights of conscripts; since 2014, the organization was declared a “foreign agent”.^50^ Valentina mELNIKOVA, the head of the organization of Soldiers' Mothers, reported in a media interview that mothers of the Russian soldiers mobilized to the war often lack information about their sons, many of whom are sent to fight in Ukraine against their will.^51^

## Conclusion

On the basis of the studied material, it is possible to say that the ideas of “traditional values” and “familism” were supported and distributed by several different actors, only some of whom were closely associated with the ROC. Even if there were more differences than similarities in the beginning, by the mid-2010s, these actors mainly agreed on the concept of motherhood based on the family with several children, non-acceptance of abortion, the nuclear family as the center of educational and moral choices, and with the woman–mother who sees her maternity as her most important social responsibility. The narrowing distance between these actors happened with the open support of the state, with the aim of controlling the reproductive capacities of women's bodies and social reproduction to strengthen Russia's geopolitical position in the world.

The new values were diffused through a complex network of actors, an important part of which consisted of the state-controlled civil society organizations, made possible in the context of the ban on international funding and cooperation for NGOs and a huge propagandistic campaign about “Western” human rights and liberal values damaging “national security”. State-controlled women's organizations have played a prominent role among these actors, mobilizing women to be “good mothers” according to the interests of the state and the nation. With the help of some Soviet rhetoric on “happy motherhood”, these ideas became framed as the slogans and values that could be shared by the broader female population of Russia (not necessarily just conservative and Orthodox). Thus, the ideology of “good mothers” and “strong families” could be used not only against the rights of queer people and women but also, as the latest developments show, for securing social support for the war.

## Author contributions

The author confirms being the sole contributor of this work and has approved it for publication.

## Footnotes

^1^See, for example, women in Dagestan protesting against the mobilization of their sons. https://edition.cnn.com/2022/09/26/europe/russia-dagestan-protests-mobilization-intl-hnk/index.html (accessed September 2, 2023).

^2^All the translations of the names of organizations, titles of the documents, or short quotes from Russian to English are mine.

^3^The monthly allowance can be paid in cases where family income is not over the established limit.

^4^https://www.vedomosti.ru/society/news/2021/11/30/898265-demografiyu-osnovnoi-problemoi (accessed September 2, 2023).

^5^For more on the achievements and contradictions of the realization of this program, see Kulmala et al. (2021).

^6^See https://www.bbc.com/russian/news-62577646; http://duma.gov.ru/news/51772/ (accessed September 2, 2023).

^7^The media present them as threatening the school learning of all children through a low level of knowledge of the Russian language and showing a low level of preparation for school.

^8^Antonov “Chto nuzhno sdelat dlya uvelicheniia kolichestva mnogodetnykh semei v Rossii” Audiofile http://www.proektnoegosudarstvo.ru/publications/0146/20.12.2013 (accessed September 2, 2023).

^9^For example, “traditional values” are often used by Putin in different contexts (Johnson et al., 2021).

^10^“Krepkaia semia—silnaia Rossiia”—slogan of the organization “Materi Rossii” https://xn–e1abcgakjmf3afc5c8g.xn–p1ai/communication/forums/family/semya-lyubov-i-vernost-samye-velikie-chelovecheskie-tsennosti/ (accessed September 2, 2023).

^11^https://pk-semya.ru/ (accessed September 2, 2023).

^12^https://pk-semya.ru/meropriyatiya.html. The website also has links to several other educational and cultural events; for example, the educational week “For Life – Mikhailov days” (held in 2014) or interviews with Galina Alekseeva, organizer of the short films festival “Family of Russia” in 2013 (accessed September 2, 2023).

^13^https://conkurs-cm.fap.ru/ (accessed September 2, 2023).

^14^https://conkurs-cm.fap.ru/contest/2429?pgstatic=about (accessed September 2, 2023).

^15^https://www.pravoslavie.ru/41/ (accessed September 2, 2023).

^16^https://foma.ru/cat/semja (accessed September 2, 2023).

^17^https://foma.company.site/ (accessed September 2, 2023).

^18^Following religious understanding about life, the book sees a fetus as a child.

^19^https://360tv.ru/tekst/obschestvo/sojuz-zhenschin-rossii/ (accessed September 2, 2023).

^20^https://www.bbc.com/news/blogs-news-from-elsewhere-53319505 (accessed September 2, 2023).

^21^15.09.2020 https://wuor.ru/news/11432-universitet_materinstva; https://wuor.ru/news/3422-materinstvo_put_k_schastyu/ (accessed September 2, 2023).

^22^10.12.2020 https://wuor.ru/news/13185-nagrada_za_materinstvo (accessed September 2, 2023).

^23^See, for example, the Volgograd region, 10.12.2020 https://wuor.ru/news/13185-nagrada_za_materinstvo/ (accessed September 2, 2023). 30.11.2015 https://xn–e1abcgakjmf3afc5c8g.xn–p1ai/communication/forums/family/den-materi-glavnoe-slovo-na-svete/; the Union of Women's Forces was also the initiator of the special premium for mother heroes. 28.05.2020 https://xn–e1abcgakjmf3afc5c8g.xn–p1ai/communication/forums/family/mamina-slava-istoriya-nagrady/?commentId=263302&sphrase_id=41125200#263302 (accessed September 2, 2023).

^24^19.10.2018 https://wuor.ru/news/3248-seminar-trening_psihologicheskoe_konsultirovanie_zhenschin_v_situatsiyah_reproduktivnogo_vibora/ (accessed September 2, 2023).

^25^It was created on the day of Love and Fidelity in 2017. https://xn–e1abcgakjmf3afc5c8g.xn–p1ai/about/ (accessed September 2, 2023).

^26^https://materirossii.ru/vpn/ (accessed September 2, 2023).

^27^https://www.vneshtorgclub.com/index.php?option=com_content&view=article&id=367:woman-union&catid=50:strategic&Itemid=220 (accessed September 2, 2023).

^28^https://xn–e1abcgakjmf3afc5c8g.xn–p1ai/events/10384/ (accessed September 2, 2023).

^29^https://materirossii.ru/semya-eto-samoe-glavnoe-chto-xochet-imet-v-svoej-zhizni-kazhdyj-iz-nas/ (accessed September 2, 2023).

^30^For example, in her recent interview (8.08.2023), Petrenko insisted that it does not correspond to “our traditions” to live together and have children without being officially married. Thus, the education of boys and girls should contribute to preventing it. https://vk.com/video/@vod.materi.rossii?z=video709655904_456239071%2Fpl_709655904_-2 (accessed September 2, 2023).

^31^Lakhova in YouTube interview “Strategia v interesakh zhenshchin v deistvii”, taken before EWF in October 2021—https://www.youtube.com/watch?v=2XlRifCLcjY (accessed September 2, 2023).

^32^See, for example, the filmed interview with Lakhova, head of WUOR, from October 2021.

^33^Natsionalnaia strategiia deitviia v interesakh zhenshchin, 2017-2022, https://zhit-vmeste.ru/gosprogramma-dostupnaya-sreda/normativnye-pravovye-akty/akty-pravitelstva-rossiyskoy-federatsii/119374/ (accessed September 2, 2023).

^34^https://smotrim.ru/audio/1480346 16.04.2014 “Intensivnost migratsii takova chto pol Rossii iz priezhzhikh i ikh potomkov—eto realno” (accessed February 2022).

^35^https://www.wuor.ru/news/pravilno-vyyti-zamuzh-v-korkino-dvorets-brakosochetaniya-prevratilsya-v-shkolu-nevest-103415/ (accessed September 2, 2023).

^36^According to Antonov, there is a direct connection between parents bringing their children to kindergartens and leaving them there as “suitcases” and then, later, adult children leaving their parents in homes for elderly people. https://rg.ru/2013/11/19/reg-cfo/antonov.html (accessed September 2, 2023).

^37^https://shamolina.ru/tag/%d1%80%d0%b0%d1%81%d1%88%d0%ba%d0%be%d0%bb%d0%b8%d0%b2%d0%b0%d0%bd%d0%b8%d0%b5/ (accessed February 1, 2022). See also https://shamolinadotru.wordpress.com/about/ (accessed September 2, 2023).

^38^The pamphlet of the pro-life festival in Penza in 2012 included a picture of a male bearded activist with a baby (ppp online; for more about this festival, see http://orskeparh.ru/index_old.php?content=news&id=346), while *Foma* published an article, “How do priests educate their children”, where the interviewed priests demonstrate a lot of interest in bringing up their children as moral beings https://foma.ru/ottsyi-o-detyah-kak-svyashhenniki-vospityivayut-svoih-detey.html. (accessed September 2, 2023).

^39^https://www.pravoslavie.ru/57817.html 2012 (accessed September 2, 2023).

^40^https://xn–e1abcgakjmf3afc5c8g.xn–p1ai/communication/forums/family/semya-lyubov-i-vernost-samye-velikie-chelovecheskie-tsennosti/ (accessed September 2, 2023).

^41^http://msk-news.net/politics/2022/07/18/280507.html (accessed September 2, 2023).

^42^https://www.theguardian.com/world/2023/jul/24/vladimir-putin-signs-law-banning-gender-changes-in-russia (accessed September 2, 2023).

^43^https://foma.ru/tag/situacija-na-ukraine (accessed September 2, 2023).

^44^https://foma.ru/cerkov-peredala-bezhencam-i-postradavshim-v-zone-konflikta-uzhe-svyshe-2-000-tonn-pomoshhi.html; https://foma.ru/cerkovnomu-shtabu-pomoshhi-bezhencam-v-moskve-nuzhny-produkty.html (accessed September 2, 2023).

^45^https://eawf.ru/news/novosti-soveta-ezhf/; https://eawf.ru/news/novosti-soveta-ezhf/programmu-zhenshchina-lider-vstrechayut-v-uralskom-federalnom-okruge/ (accessed September 2, 2023).

^46^https://www.wuor.ru/news/pomozhem-detyam-donbassa-odna-iz-samykh-masshtabnykh-gumanitarnykh-aktsiy-rossii-112765/ (accessed September 2, 2023).

^47^https://www.wuor.ru/news/natalya-korshunova-donbass-vnov-stanovitsya-chastyu-bolshogo-russkogo-mira-102176/ (accessed September 2, 2023).

^48^https://wuor.ru/news/v-altayskom-krae-prazdnovali-vossoedinenie-kryma-s-rossiey-88638/ (accessed September 2, 2023).

^49^https://wuor.ru/news/obrashchenie-soyuza-zhenshchin-rossii-87792/ (accessed September 2, 2023).

^50^https://ksmrus.ru/ (accessed September 2, 2023).

^51^https://rightlivelihood.org/news/willing-or-not-they-send-everyone-considered-necessary-says-representative-of-russian-soldiers-mothers/ (accessed September 2, 2023).
